# Dietary *Bacillus subtilis* Improves Growth Performance, Digestive Enzyme Activity, Antioxidant and Inflammatory Responses, and Gut Microbiota in Juvenile GIFT (*Oreochromis niloticus*)

**DOI:** 10.3390/ani16131942

**Published:** 2026-06-23

**Authors:** Qin Zhang, Nuoyun Qin, Daidi Xu, Zhichang He, Lanqian Xiang, Miao Zhou, Ziyang Yuan, Tong Tong, Yongqiang Liu, Zhongbao Guo

**Affiliations:** 1 Guangxi Key Laboratory for Polysaccharide Materials and Modifications, Guangxi Marine Microbial Resources Industrialization Engineering Technology Research Center, School of Marine Sciences and Biotechnology, Guangxi Minzu University, 158 University Road, Nanning 530008, China; zhangqin@gxmzu.edu.cn (Q.Z.); qinnuoyun@stu.gxmzu.edu.cn (N.Q.); xudaidi@stu.gxmzu.edu.cn (D.X.); xianglanqian@stu.gxmzu.edu.cn (L.X.); zhoumiao@stu.gxmzu.edu.cn (M.Z.); yuanziyang@stu.gxmzu.edu.cn (Z.Y.);; 2 Guangxi Key Laboratory for Aquatic Genetic Breeding and Healthy Aquaculture, Guangxi Academy of Fishery Science, 8 Qingshan Road, Nanning 530021, China; jackson_bourne@outlook.com

**Keywords:** probiotic supplementation, aquaculture nutrition, redox regulation, intestinal health, microbial community

## Abstract

This study investigated the effects of dietary *Bacillus subtilis* supplementation on growth performance, digestive enzyme activity, expression of antioxidant- and inflammation-related genes, and gut microbiota in juvenile GIFT (*Oreochromis niloticus*). The juvenile GIFT (total 540, initial body weight: 16.17 ± 1.32 g) were randomly divided into six groups and fed diets containing 0, 1 × 10^7^, 1 × 10^8^, 1 × 10^9^, 1 × 10^10^, or 1 × 10^11^ CFU/kg *B. subtilis* for 60 days. Compared with the control group (0 CFU/kg *B. subtilis*), dietary *B. subtilis* significantly improved final body weight, weight gain rate, specific growth rate, and daily growth index and reduced feed conversion ratio (*p* < 0.05). Supplementation also significantly increased intestinal amylase, lipase, and trypsin activities (*p* < 0.05). In addition, the expression of several antioxidant-related genes was upregulated, whereas pro-inflammatory gene expression was generally downregulated in the intestine, liver, and head kidney. Gut microbiota analysis indicated that *B. subtilis* supplementation altered community composition, increased Firmicutes’ abundance, and reduced Proteobacteria abundance. Among all supplementation levels evaluated, the diet containing 1 × 10^9^ CFU/kg B. *subtilis* produced the most favorable overall beneficial effects under the conditions of the present study.

## Simple Summary

The use of safe feed additives is increasingly important for improving fish health and supporting sustainable aquaculture. This study investigated the effects of dietary *Bacillus subtilis* supplementation on growth performance, digestive enzyme activity, expression of antioxidant- and inflammation-related genes, and gut microbiota in juvenile GIFT (*Oreochromis niloticus*). After 60 days, supplemented fish grew better, used feed more efficiently, and showed stronger digestive and antioxidant capacity, with lower inflammatory responses. Their intestinal bacterial community also shifted in a more favorable direction. Among the tested diets, the 1 × 10^9^ CFU/kg supplementation level gave the most consistent benefits. These findings support the use of *Bacillus subtilis* as a practical dietary additive in tilapia farming.

## 1. Introduction

The Genetically Improved Farmed Tilapia (GIFT, *Oreochromis niloticus*), characterized by rapid growth, genetic stability and strong environmental adaptability, has become a cornerstone species in China’s aquaculture industry [1]. In recent years, demand for tilapia in international markets has continued to grow, and its share of the global freshwater fish trade is second only to that of trout [2]. The development of the tilapia industry can enhance food security, nutritional value, and the income of aquaculture farmers, thereby improving the well-being of people in developing countries [3]. However, intensive, high-density aquaculture practices have been accompanied by problems such as water quality deterioration, the overuse of antibiotics, and damage to the aquaculture ecosystem [4]. This not only leads to an imbalance in the gut microbiota of farmed animals and a weakened immune system, but also creates safety hazards, for example, drug residues and the proliferation of drug-resistant bacteria, severely impacting the production and economic efficiency of tilapia farming [5]. Therefore, finding environmentally friendly and safe solutions has become an urgent priority for the industry.

Probiotics have functions such as promoting growth, enhancing disease resistance, and regulating gut health and is considered an important approach to alleviating the challenges faced by the aquaculture industry [6]. *Bacillus subtilis* is a Gram-positive bacterium characterized by high stability, a wide pH tolerance range, heat resistance, and the ability to form spores to survive adverse conditions; it is used in the aquaculture industry [7,8]. Studies have shown that *Bacillus* species play a significant role in enhancing growth performance, disease resistance, and immune function; reinforcing intestinal epithelial barrier integrity; modulating the composition and functionality of the gut microbiota; and improving aquaculture water quality [9,10,11]. First, after *Bacillus* species colonize the gut, the secondary metabolites they secrete help the host utilize nutrients, and The short-chain fatty acids (SCFAs) produced stimulate the growth of the intestinal epithelium in aquatic animals such as Chinese perch (*Siniperca chuatsi*) and tilapia, thereby improving nutrient utilization and growth performance [12,13]. Second, *Bacillus* produce antimicrobial peptides and secondary metabolites that maintain intestinal health in Rohu carp (*Labeo rohita*) by enhancing immune regulation and inhibiting the proliferation of harmful bacteria, thereby effectively improving the fish’s resistance to *Aeromonas hydrophila* [8].

The gut is a vital organ for digestion, metabolism, and immunity in fish, whose health directly affects the overall health of the fish [14]. During the early juvenile stage, the intestinal structure of fish is not yet fully developed, and the gut microbiota has not yet established a stable community structure, thus the composition of the microbiota is easily influenced by the rearing environment and feed composition [15]. The gut microbiota plays an indispensable role in host physiological processes, including dietary digestion, nutrient absorption, and defense against exogenous pathogens [16].

Currently, most research on *Bacillus subtilis* in tilapia aquaculture focuses on its effects on host metabolism and immune function and its contribution to water quality maintenance [17,18]. However, research on the effects of *Bacillus subtilis* on the growth performance, immune function, and gut microbiota composition of tilapia is relatively limited. As a feed additive, *Bacillus subtilis* plays a crucial role during the juvenile stage that a critical period of growth and supplementing this strain during this stage may have a significant impact on early development, immune function, and disease resistance [19]. Therefore, this study aims to evaluate the effects of different levels of *Bacillus subtilis* dietary on growth performance, digestive enzyme activity, antioxidant and inflammatory gene expression, and gut microbiota composition in juvenile tilapia. The results will provide a scientific basis for determining the optimal addition level of *Bacillus subtilis* dietary for juvenile GIFT and for exploring methods related to microbial community regulation.

## 2. Materials and Methods

### 2.1. Experimental Materials

The *Bacillus subtilis* strain (GXF1608) used in this experiment is a strain preserved by the Key Laboratory of Conservation and Utilization of Marine Biological Resources at Guangxi Minzu University. The strain was isolated from mangrove soil using the method described by Zhang et al. [20] and identified as *Bacillus subtilis* through 16S rRNA gene sequence clustering analysis that exhibits rapid growth and high production of alkaline protease and is stored at −80 °C [21]. For bacterial isolation, cryopreserved strains were streaked onto LB agar plates, and incubation was maintained at 28 °C for 48 h. Subsequently, a single dominant colony was selected under aseptic conditions and transferred into 10 mL of LB liquid medium. The culture was incubated on a shaking incubator (140 rpm, 28 °C) for 10 h. The resulting initial bacterial suspension was then transferred under aseptic conditions to 300–500 mL of LB liquid medium and incubated continuously on a shaking incubator (140 rpm, 28 °C) for 48 h. The bacterial cells were harvested through refrigerated centrifugation (4000× *g*, 15 min, 4 °C) and subsequently lyophilized to obtain a stable probiotic preparation. The resulting lyophilized powder has a live cell concentration of no less than 10^11^ CFU/g and is stored at −80 °C.

The present investigation was carried out on the basis of earlier studies and safety assessment outcomes [22]. In summary, 120 juvenile GIFT (14.22 ± 1.58 g) were immersed in a *Bacillus subtilis* suspension at final concentrations of 0, 10^5^, 10^7^, 10^9^, and 10^11^ CFU/mL, with three replicate groups at each concentration, each containing 10 fish. After performing a complete water change every 24 h, the *Bacillus subtilis* suspension was reintroduced into each aquarium, and the physical condition and mortality rate of the juvenile GIFT and observed for 14 days. Experimental results have shown that *Bacillus subtilis* does not cause death (survival rate: 100%).

### 2.2. Experimental Diet

The experimental diets were prepared following the methods described by Ji et al. [23] and Xiong et al. [24]. All feed ingredients were purchased from Nanning Tongwei Feed Co., Ltd. (Nanning, China) and are of food-grade quality. Each ingredient was weighed accurately as specified by the formula listed in Table 1. With fish oil and soybean oil excluded, all solid ingredients were subjected to fine grinding and subsequent 60-mesh sieving treatment. During the preparation process, the bacterial strain is first mixed with soybean meal and then incorporated into the basal diet to achieve final concentrations of G0 (0), G1 (1 × 10^7^), G2 (1 × 10^8^), G3 (1 × 10^9^), G4 (1 × 10^10^), and G5 (1 × 10^11^ CFU/kg) diet. Then it was mixed further with the other ingredients in a drum mixer for 15 min, and finally about 40% water was added to thoroughly blend all the ingredients. The mixture was shaped by hand into small pellets, which were then passed through a sieve to produce feed pellets of uniform size, ranging from 1.00 to 1.50 mm in diameter. The prepared feed was dried at 30 °C until the moisture content dropped below 100 g/kg. Finally, the dried feed is packaged in sterile plastic bags and stored at −20 °C for future use. To ensure bacterial viability during the feeding trial, fresh diets were prepared every four days. The viable bacterial count of each diet batch was determined using the plate-counting method immediately after preparation [25]. The measured bacterial concentrations in the experimental diets were G1 (0.89 ± 0.20) × 10^7^, G2 (0.95 ± 0.13) × 10^8^, (1.12 ± 0.08) × 10^9^, (0.98 ± 0.18) × 10^10^, and (1.09 ± 0.23) × 10^11^ CFU/kg.

### 2.3. Experimental Fish and Culture

The experiment with juvenile GIFT was approved by the Biomedical Ethics Committee of Guangxi Minzu University in Nanning, China (Approval No.: GXMZU-2022-008), and the 1000 juvenile GIFT were obtained from Guangxi Fry Breeding Base in Nanning, China. After being disinfected with a 20 mg/L potassium permanganate solution, the fry acclimated to the environment in the grow-out pond for 15 days. During the acclimatization period, the rearing tanks used a recirculating aquaculture system with water temperature of 27 ± 1 °C, pH od 7.5–8.0, ammonia nitrogen od < 0.5 mg/L, nitrite content of <0.05 mg/L and dissolved oxygen of ≥7.5 mg/L. The fish were fed the control diet three times daily at 9:00 a.m., 2:00 p.m., and 7:00 p.m., with the daily feed intake amounting to 5% of the total wet body weight of the fish. The excreta was cleaned from the aquaculture system every morning before feeding, and 1/3 of the total water volume was replaced. After the acclimatization period, the juvenile GIFT were subjected to a 24 h fasting regimen. We randomly selected 540 healthy fingerlings with uniform coloration and body shape and divided them into 6 groups, each with 3 replicates, for a total of 18 rearing tanks. Each tank had a volume of 720 L (length × width × height: 150 × 60 × 80 cm) and housed 30 GIFT fingerlings. During the experimental period, the fish were cultured in the same aquaculture system as used for acclimatization and were fed three times daily at 9:00 a.m., 2:00 p.m., and 7:00 p.m. The control group’s feed did not contain *Bacillus subtilis*, while the feed for the other experimental groups contained 1 × 10^7^, 1 × 10^8^, 1 × 10^9^, 1 × 10^10^, and 1 × 10^11^ CFU/kg of *Bacillus subtilis*, respectively. During the 60-day feeding trial, the daily feed intake for the fish was equivalent to 5% of their wet body weight. Every 7 days, 6 fish are randomly selected from each tank and weighed to calculate the average weight. The weekly daily feeding amount is then calculated using the formula: average weight × number of fish per tank × 5%. These measurements were used exclusively to adjust the daily feeding ration. Feed intake was recorded throughout the experiment and used for FCR calculation.

### 2.4. Sample Collection

At the end of the 60 day feeding trial, juvenile GIFT were fasted for 24 h prior to sample collection. A total of 18 tanks were used in the experiment, with three replicate tanks assigned to each dietary treatment. For growth performance, digestive enzyme activity, and gene expression analyses, nine fish were randomly selected from each treatment group (three fish from each replicate tank). For gut microbiota analysis, six fish were randomly selected from each treatment group (two fish from each replicate tank). Intestinal samples collected from fish within the same tank were pooled and treated as one biological replicate for microbial community analysis. The samples were anesthetized with 0.2 g/L ethyl 3-aminobenzoate mesylate (MS-222, Adamas, Shanghai Adamas Reagent Co., Ltd., Shanghai, China). Body weight and total length were measured prior to dissection. Subsequently, the samples were collected from the head kidney, whole intestine, spleen, and liver. The entire dissection was performed on ice. Following collection, all samples were placed in labeled sample bags and rapidly frozen in liquid nitrogen. Once sampling had been completed, the samples were stored in an ultra-low-temperature freezer at −80 °C pending subsequent analysis.

### 2.5. Growth Performance Measurement

The methods for calculating survival rate (SR, %), weight gain rate (WGR, %), specific growth rate (SGR, %/day), daily growth index (DGI, %/day), and feed conversion ratio (FCR) are as follows:

$$SR(\%)=100\times \frac{final\;number\;of\;fish}{initial\;number\;of\;fish}$$

$$WGR(\%)=100\times \frac{final\;body\;weight\;(g)-initial\;body\;weight\;(g)}{initial\;body\;weight\;(g)}$$

$$SGR(\%/d)=100\times \frac{\left[ln\;(final\;body\;weight)\;(g)]-[ln\;\left(initial\;body\;weight\right)\left(g\right)\right]}{days}$$

$$DGI(\%/d)=100\times \frac{{\left[(final\;body\;weight)\;(g)\right]}^{1/3}-{\left[(initial\;body\;weight)\;(g)\right]}^{1/3}}{days}$$

$$FCR=\frac{feed\;intake\;(g)}{final\;body\;weight\;(g)-initial\;body\;weight\;(g)}$$

### 2.6. Digestive Enzyme Activities

Based on the method described by Liu et al. [26], the activities of α-amylase, lipase, and trypsin in the whole intestines of juvenile GIFT were determined using the α-amylase assay kit (C016-1-1), the lipase assay kit (A054-2-1), and the trypsin assay kit (A080-2). All assay kits were supplied by the Nanjing Jiancheng Institute of Biological Engineering (Nanjing, China).

### 2.7. Gene Expression

We determined the expression of antioxidant-related genes in the intestines and livers of juvenile GIFT, including superoxide dismutase (*sod*), catalase (*cat*), glutathione S-transferase (*gst*), glutathione peroxidase (*gpx*), and nuclear factor E2-related factor 2 (*nrf2*). Determination of genes associated with inflammatory responses in the head kidneys, liver, spleen, and intestines of juvenile GIFT with tumor necrosis factor (*tnf-α*), *il-1β* (interleukin-1β), transforming growth factor-β1 (*tgf-β1*), interleukin-6 (*il-6*), interleukin-8 (*il-8*), and interleukin-10 (*il-10*). The specific methods are described in Zhang et al. [27]. For RT-qPCR analysis, *β-actin* was employed as the internal reference gene. The primer pairs were designed using Primer Premier 6.0 based on tilapia mRNA sequences available in the National Center for Biotechnology Information (NCBI) database. The primers were synthesized by Sangon Biotech (Shanghai) Co., Ltd. In Shanghai, China; see Table 2 for details.

First, total RNA was initially extracted from liver, spleen, head kidney, and intestinal tissue samples of juvenile GIFT with the Takara MiniBEST Universal RNA Extraction Kit (Universal Type; Takara Biomedical Technology Co., Ltd., Beijing, China). For detailed instructions, please refer to the kit manual. A total of 1000 ng of RNA was used for cDNA synthesis with the PrimeScript™ RT Master Mix (Perfect Real-Time) reverse transcription kit (Takara Biomedical Technology Co., Ltd., Beijing, China). The reaction mixture was prepared in a final volume of 50 μL. The synthesized cDNA was used as the template for subsequent quantitative analysis. Quantitative real-time PCR was performed using TB Green^®^ Premix Ex Taq™ II (Tli RNaseH Plus; Takara Bio Inc., Dalian, China) on a LongGene real-time PCR system (Q2000B). Each reaction was conducted in a 20 μL volume. The analysis of the obtained primer melting curves revealed that each primer exhibited a single peak, indicating specific amplification and suitability for subsequent experimental analysis. Relative gene expression was determined using the 2^−ΔΔCt^ method [28].

### 2.8. Gut Microbiota

The collected intestinal contents sent to Guangzhou Gene Denovo Biotechnology Co., Ltd. (Guangzhou, China) for sequencing using the Illumina MiSeq PE250 high-throughput sequencing platform. All experimental steps were performed following the corresponding manufacturers’ instructions. The high-throughput sequencing results of tilapia gut bacteria were comprehensively analyzed using Omicsmart, an online real-time interactive data analysis platform provided by Gene Denovo. After obtaining raw sequencing reads, low-quality reads were first filtered and removed. Sequence assembly and subsequent filtering were then performed to cluster and generate Operational Taxonomic Units (OTUs), grouping sequences with over 97% similarity into the same OTU. Subsequently, quantitative analysis was performed on each OTU to determine the relative abundance of tag sequences across samples. Species annotation was conducted using the RDP classifier for representative OTUs. Alpha and beta diversity analyses were performed to assess differences among the six groups of tilapia gut microbial samples. Homogeneity of multivariate dispersion was evaluated prior to interpreting beta diversity differences among treatment groups. To assess whether PERMANOVA results could be influenced by unequal within-group dispersion, a permutational analysis of multivariate dispersions (PERMDISP) was performed using the ‘betadisper()’ function in the vegan package (version 2.7-5) implemented in R version 4.6.0. Statistical significance was assessed using the ‘permutest()’ function with 999 permutations. The same Bray–Curtis distance matrix used for PCoA and PERMANOVA was employed for PERMDISP analysis to ensure consistency among beta diversity analyses. PERMDISP was performed to verify the assumption of homogeneous multivariate dispersion among groups and to distinguish differences in community composition (location effects) from differences in within-group variability (dispersion effects). At the phylum and order levels of biological classification, the bacterial community structure and relative abundance of each taxonomic group within the six tilapia gut samples were meticulously analyzed. Finally, KEGG pathway prediction was performed using PICRUSt2, and functional abundance statistics for KO (KEGG Orthology) abundance were calculated to predict the potential functions of the gut microbiota.

### 2.9. Data Statistics and Analysis

All data were initially entered and sorted in Microsoft Excel 2025. Then, a one-way analysis of variance (ANOVA) was performed on the data using IBM SPSS 27 software. The Least Significant Difference (LSD) test was used to assess significant differences between groups. Statistical significance was set at *p* < 0.05, and results are expressed as mean ± standard deviation (mean ± SD). The figures were generated using GraphPad Prism 10 software.

## 3. Results

### 3.1. Growth Performance

As shown in Table 3, compared to the juvenile GIFT fed control diet (0), the survival rate (SR), final body weight (FBW), weight gain rate (WGR), specific growth rate (SGR), and daily growth index (DGI) of the juvenile GIFT fed different dietary *Bacillus subtilis* supplementation (1 × 10^7^, 1 × 10^8^, 1 × 10^9^, 1 × 10^10^, and 1 × 10^11^ CFU/kg) were significantly increased (*p* < 0.05). The highest final body weight, WGR, SGR and DGI were found in the tilapia fed 1 × 10^9^ CFU/kg *Bacillus subtilis*, respectively.

Compared to the juvenile GIFT fed control diet (0), the feed conversion ratio (FCR) of the juvenile GIFT fed different dietary *Bacillus subtilis* supplementation (1 × 10^7^, 1 × 10^8^, 1 × 10^9^, 1 × 10^10^, and 1 × 10^11^ CFU/kg) was significantly decreased (*p* < 0.05). The lowest FCR was found in the tilapia fed 1 × 10^9^ CFU/kg *Bacillus subtilis*.

### 3.2. Digestive Enzyme Activities

As shown in Table 4, the activities of intestinal α-amylase, lipase, and trypsin in juvenile GIFT fed different dietary *Bacillus subtilis* supplementation (1 × 10^7^, 1 × 10^8^, 1 × 10^9^, 1 × 10^10^, and 1 × 10^11^ CFU/kg) showed an initial increase followed by a decrease. In particular, G3 (1 × 10^9^ CFU/kg) showed a significant increase in α-amylase activity (*p* < 0.05).

### 3.3. Antioxidant Gene Expression

**Figure 1:**
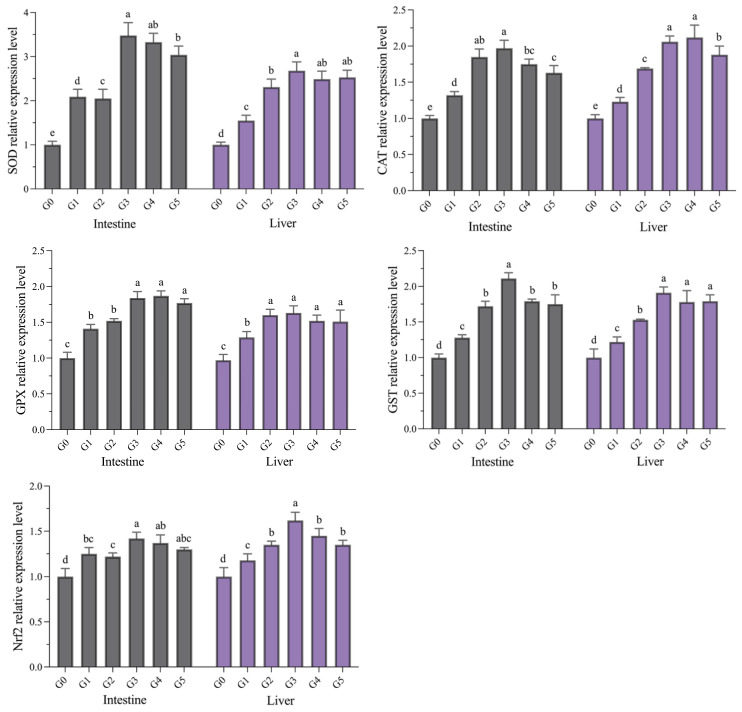
Effects of gradient concentrations of *Bacillus subtilis* on the relative expression level of superoxide dismutase (sod), catalase (cat), glutathione peroxidase (gsh-px), glutathione S-transferases (gst) and erythroid-derived 2 (nrf2) genes in the intestine and liver of the GIFT juveniles. All the data are presented as mean ± SD (*n* = 3). According to the LSD test, the mean values marked with different superscript letters in the same figure are significantly different from each other (*p* < 0.05).

In Figure 1, compared to the juvenile GIFT fed control diet (0), the *sod*, *cat*, *gst*, *gsh-px* and *nrf2* of relative expression in the intestinal of juvenile GIFT fed different dietary *Bacillus subtilis* supplementation (1 × 10^7^, 1 × 10^8^, 1 × 10^9^, 1 × 10^10^, and 1 × 10^11^ CFU/kg) were significantly increased (*p* < 0.05). The highest relative expression of *sod*, *nrf2*, *gst* and *cat* were found in the intestine of juvenile GIFT fed 1 × 10^9^ CFU/kg *Bacillus subtilis*, respectively. The highest relative expression of *gsh-px* was found in the intestinal of juvenile GIFT fed 1 × 10^10^ CFU/kg *Bacillus subtilis*.

Compared to the juvenile GIFT fed control diet (0), the *sod*, *cat*, *gst*, *gsh-px* and *nrf2* of relative expression in the liver of juvenile GIFT fed different dietary *Bacillus subtilis* supplementation (1 × 10^7^, 1 × 10^8^, 1 × 10^9^, 1 × 10^10^, and 1 × 10^11^ CFU/kg) were significantly increased (*p* < 0.05). The highest relative expression of *sod*, *nrf2*, *gst* and *gsh-px* were found in the intestine of juvenile GIFT fed 1 × 10^9^ CFU/kg *Bacillus subtilis*. The highest relative expression of *cat* was found in the intestine of juvenile GIFT fed 1 × 10^10^ CFU/kg *Bacillus subtilis*.

### 3.4. Inflammatory Gene Expression

**Figure 2:**
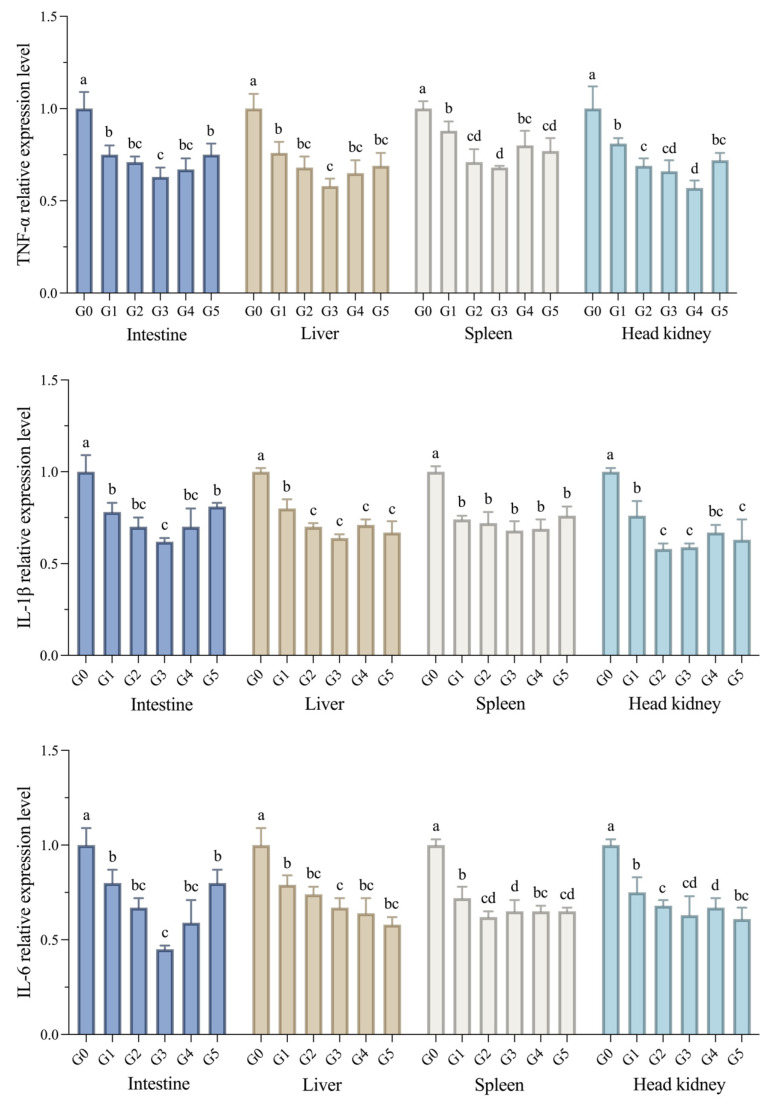

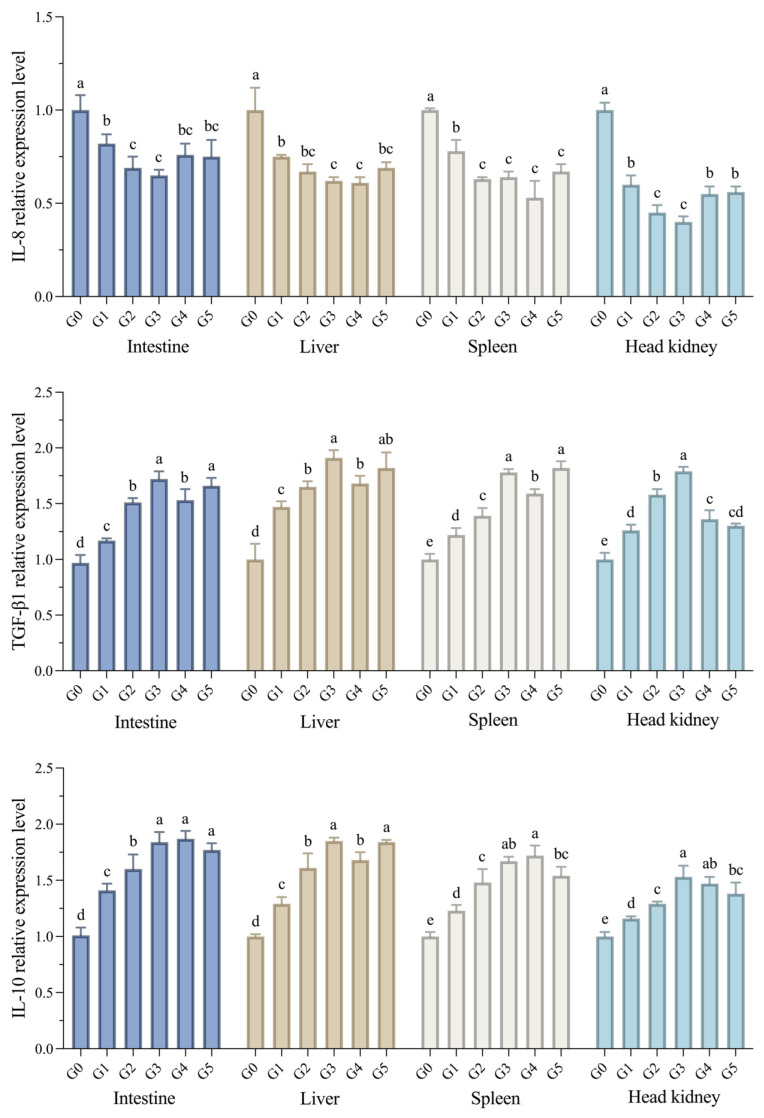
Effects of *Bacillus subtilis* on the relative expression level of tumor necrosis factor α (*tnf-α*), interleukin-1β (*il-1β*), interleukin-6 (*il-6*), interleukin-8 (*il-8*), transforming growth factor-β (*tgf-β*), and interleukin-10 (*il-10*) genes in the intestine, liver, spleen and head kidney of the tilapia GIFT juveniles. All the data are presented as mean ± SD (*n* = 3). According to the LSD test, the mean values marked with different superscript letters in the same figure are significantly different from each other (*p* < 0.05).

In Figure 2, compared to the juvenile GIFT fed control diet (0), the *tnf-α*, *il-1β*, *il-6* and *il-8* of relative expression in the different tissue of juvenile GIFT fed different dietary *Bacillus subtilis* supplementation (1 × 10^7^, 1 × 10^8^, 1 × 10^9^, 1 × 10^10^, and 1 × 10^11^ CFU/kg) were significantly downregulated (*p* < 0.05). The lower relative expression of *tnf-α* and *il-1β* were found in the intestinal, liver and spleen of juvenile GIFT fed 1 × 10^9^ CFU/kg *Bacillus subtilis*. And the lower relative expression of *il-6* and *il-8* were found in the intestine and spleen of juvenile GIFT fed 1 × 10^9^ CFU/kg *Bacillus subtilis*.

Compared to the juvenile GIFT fed control diet (0), the *il-10* and *tgf-β1* of relative expression in the different tissue of juvenile GIFT fed different dietary *Bacillus subtilis* supplementation (1 × 10^7^, 1 × 10^8^, 1 × 10^9^, 1 × 10^10^, and 1 × 10^11^ CFU/kg) were significantly upregulated (*p* < 0.05).

### 3.5. Gut Microbiota

**Figure 3:**
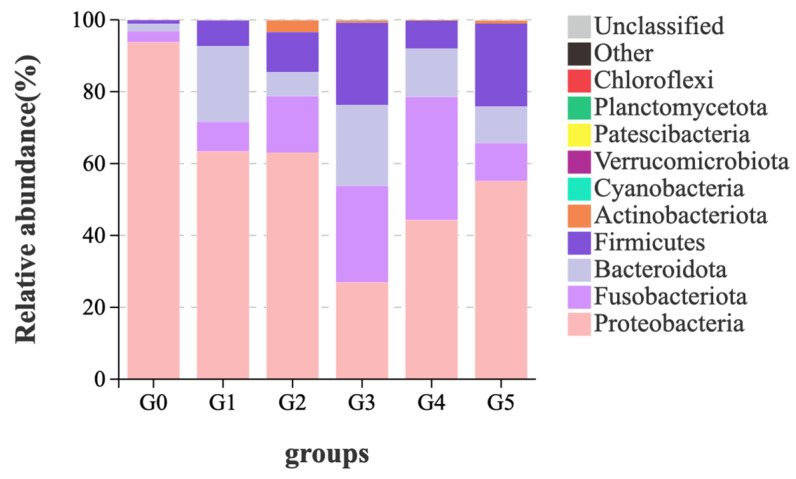
Analysis of relative abundance at the phylum level of gut microbiota.

**Figure 4:**
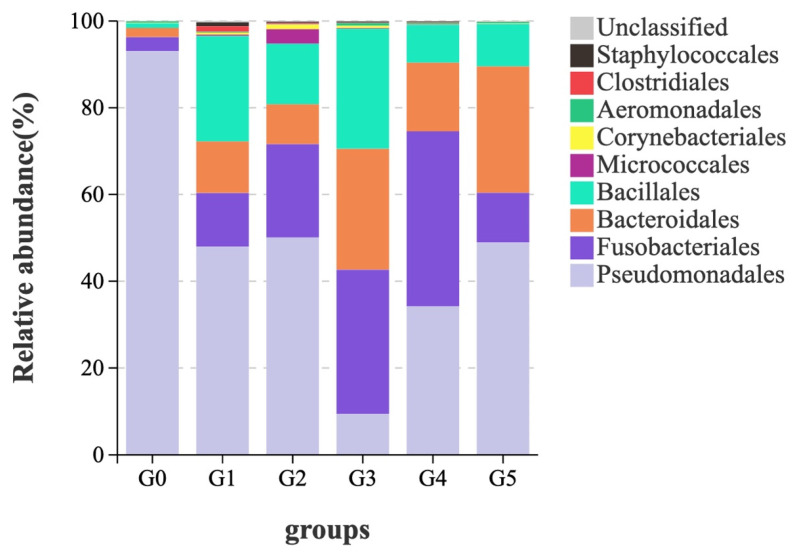
Analysis of relative abundance at the order level of gut microbiota.

Figure 3 shows the abundance at the phylum level of gut microbiota in juvenile GIFT. The intestinal microbial communities of the G0–G5 group were predominantly composed of Proteobacteria, Fusobacteriota, Bacteroidota, Firmicutes, and other bacterial phyla. The dominant phylum in Group G0 is the Proteobacteria, accounting for 93.76%; this is followed by the Fusobacteria (3.02%), Bacteroides (2.05%), and Firmicutes (1.07%). Feeding different dietary *Bacillus subtilis* supplementation reduced the proportion of Proteobacteria, with Group G3 showing the lowest proportion at 26.92%. The proportions of the Fusobacteriota, Firmicutes and Bacteroidetes, phyla all increased, with the Firmicutes and Bacteroidetes phyla reaching their highest proportions in Group G3 at 22.91% and 22.52%. Figure 4 shows the abundance at the order level of gut microbiota in juvenile GIFT. The abundance of the Pseudomonadales order decreased and Bacteroidales and Bacillales orders increased, with the Pseudomonadales order accounting for the lowest proportion (7.59%) in Group G3 following the addition of *Bacillus subtilis*.

**Figure 5:**
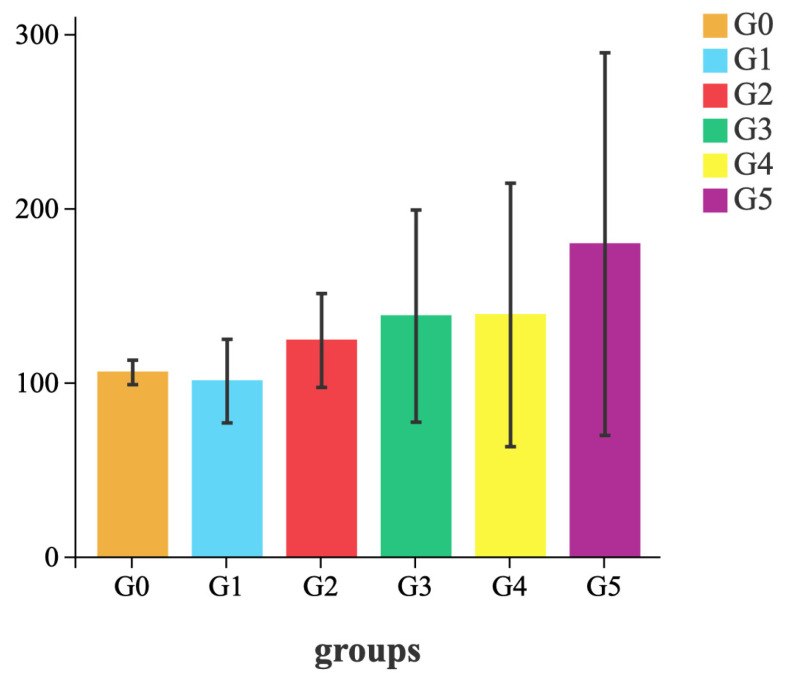
Alpha-diversity (observed species, Sob) index of gut microbiota in juvenile GIFT.

**Figure 6:**
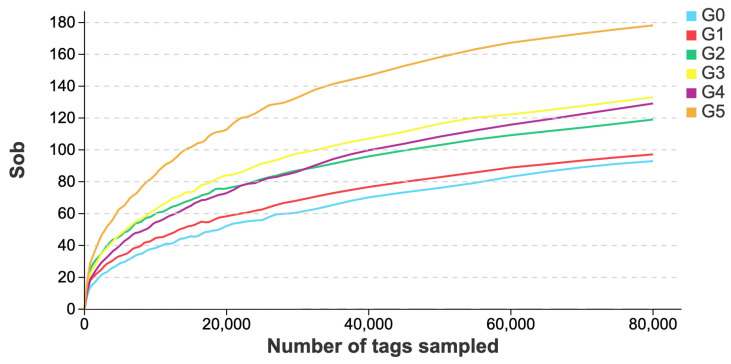
Rarefaction curves of gut microbiota in juvenile GIFT based on the observed species (Sob) index.

**Figure 7:**
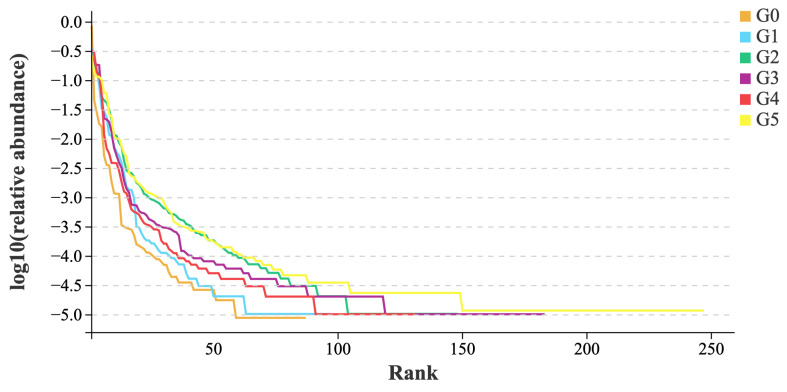
Rank–abundance curves of gut microbiota in juvenile GIFT.

**Figure 8:**
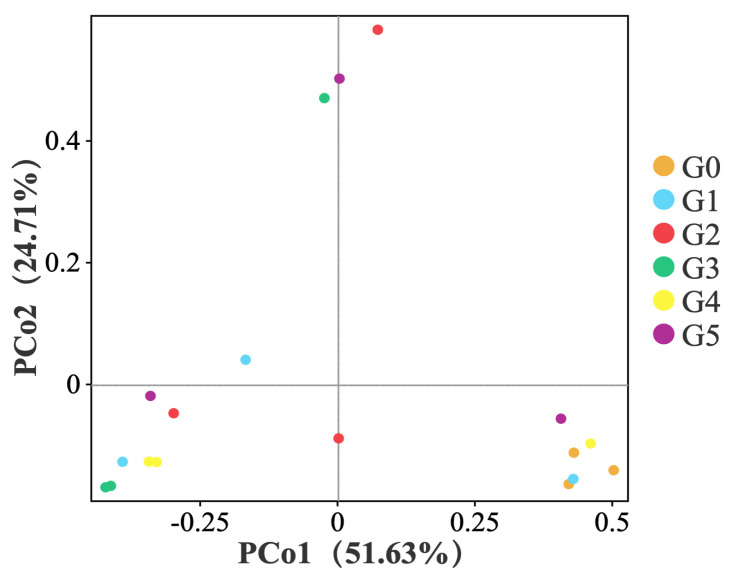
Principal coordinate analysis (PCoA) of gut microbial communities based on Bray–Curtis dissimilarity. Each point represents one intestinal sample. A total of 18 samples were analyzed—six treatment groups (G0–G5) with three biological replicates per group.

The observed species (Sobs) index was used to evaluate microbial richness in the gut microbiota of juvenile GIFT (Figure 5). The Sobs values showed an increasing trend with increasing dietary *Bacillus subtilis* supplementation. The highest richness was observed in the G5 group, whereas the lowest richness was detected in the G1 group. The rarefaction analysis demonstrated that the number of observed OTUs increased rapidly at low sequencing depths and gradually approached saturation as sequencing depth increased (Figure 6). The rank–abundance curves further illustrated the richness and evenness of the microbial communities (Figure 7). The curves of the *B. subtilis*-supplemented groups generally extended farther along the *x*-axis than that of the control group, suggesting a tendency toward greater species richness. In addition, the slopes of the curves were relatively gradual, indicating a relatively even distribution of taxa within the intestinal microbial communities. Principal coordinate analysis (PCoA) of intestinal microbial communities based on Bray–Curtis dissimilarity (Figure 8). PCo1 and PCo2 explained 51.63% and 24.71% of the total variation, respectively. Each point represents an individual sample. Although samples exhibited partial clustering according to dietary treatment, the PERMANOVA analyses indicated that the differences in overall community structure among groups were not statistically significant (*p* > 0.05). The PERMDISP analysis based on Bray–Curtis distances showed no significant differences in multivariate dispersion among treatment groups (F = 1.1181, *p* = 0.387, 999 permutations) (Figure S1).

**Figure 9:**
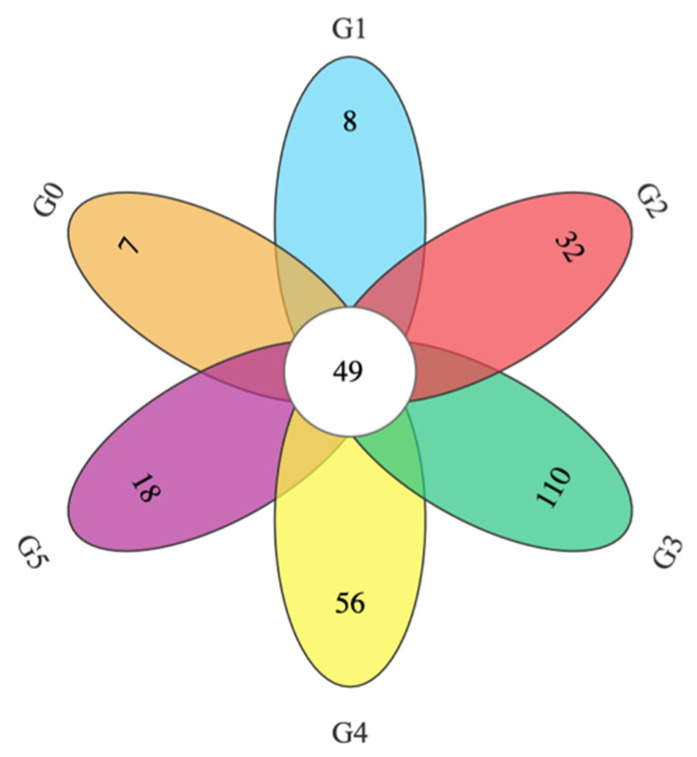
Comparison of OTUs of gut microbiota in juvenile GIFT.

**Figure 10:**
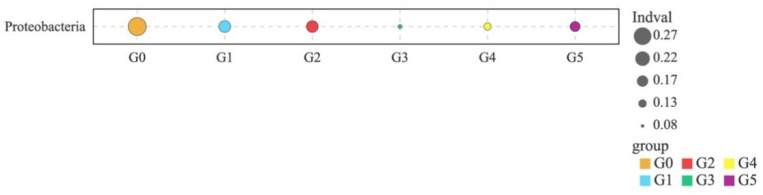
Indicator analysis of gut microbiota in juvenile GIFT.

Figure 9 shows the unique OTU number was greater in groups (G1–G5) than in the control group (G0), where only seven unique OTUs were observed. The G3 had the highest number of unique OTUs, at 110, which was higher than that of the control group and the other experimental groups; the control group and the experimental groups shared 49 OTUs. As shown in Figure 10, the indicator analysis identified biomarkers that distinguish between different groups. In the control group (G0), the Indval value for the Proteobacteria phylum was 0.27. Following the addition of *Bacillus subtilis*, the Indval values for the Proteobacteria phylum decreased across all groups, with the lowest value observed in group G3 at 0.07.

**Figure 11:**
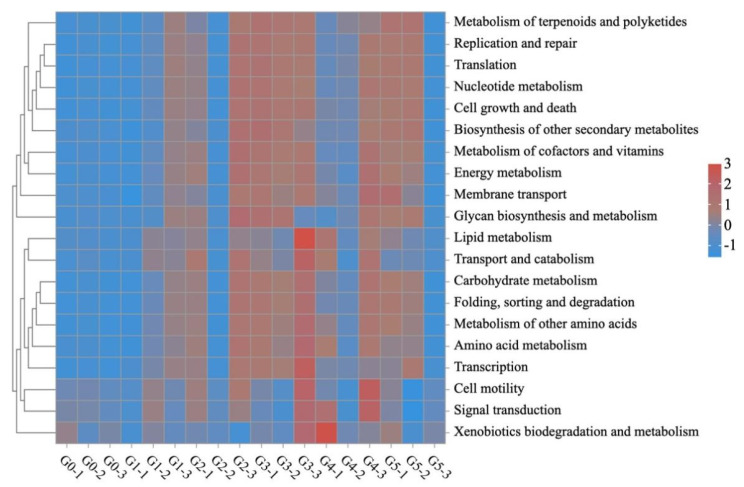
Heatmap of PICRUSt2-predicted functional abundance at KEGG pathway Level 2 in the intestinal microbiota of juvenile GIFT. The color gradient is correlated with KEGG functional abundance.

**Figure 12:**
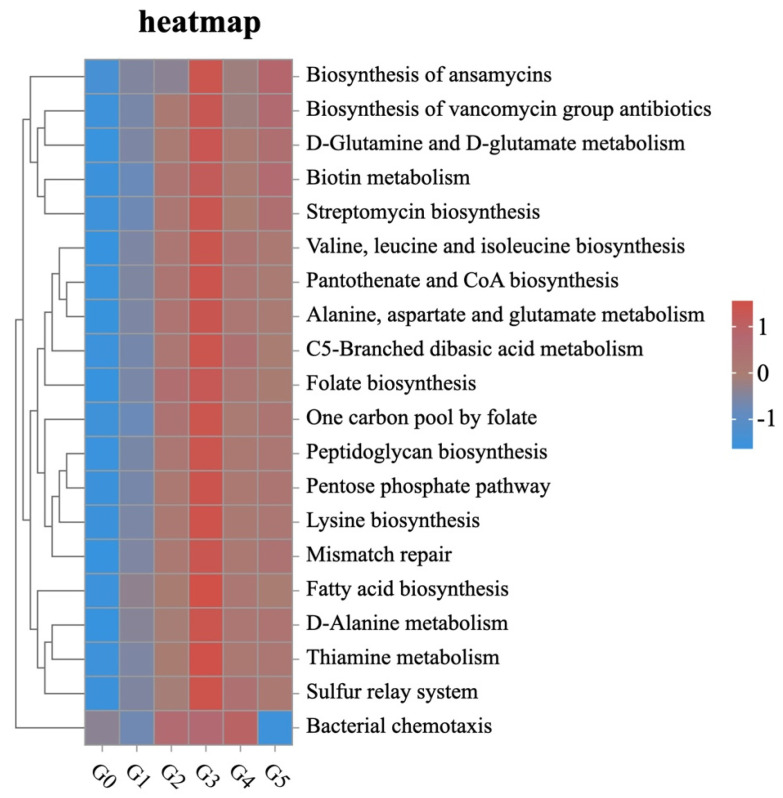
Heatmap of PICRUSt2-predicted functional abundance at KEGG pathway Level 3 in the intestinal microbiota of juvenile GIFT. The color gradient is correlated with KEGG functional abundance.

As shown in Figure 11, the predicted relative abundances of several KEGG Level 2 pathways, including lipid metabolism, cofactor and vitamin metabolism, membrane transport, and signal transduction, varied among the experimental groups following dietary *Bacillus subtilis* supplementation. Notably, the predicted relative abundance of the lipid metabolism pathway was higher in Group G3 than in Group G0. At KEGG Level 3 (Figure 12), pathways related to amino acid biosynthesis, cofactor metabolism, and vitamin metabolism showed higher predicted relative abundances in Groups G3 and G4 compared with the control group. In addition, the predicted relative abundance of energy metabolism-related pathways was higher in Group G3, whereas the bacterial chemotaxis pathway exhibited a lower predicted relative abundance in Group G5.

## 4. Discussion

### 4.1. Effects of Bacillus subtilis on the Growth Performance

In this study, dietary *Bacillus subtilis* supplementation significantly enhanced the inal weight, WGR, SGR and DGI of juvenile GIFT, while significantly reducing the FCR, with the group G3 (1 × 10^9^ CFU/kg) performing the best. This indicates that *Bacillus subtilis*, when used as a feed additive, may contribute to improve the growth performance and lower feed conversion ratio in juvenile GIFT under the present experimental conditions. This finding is generally consistent with existing studies on aquatic animals. Eissa et al. [29] found that feeding red tilapia a diet supplemented with 4 g/kg of the probiotic *Bacillus subtilis* improved their overall health and enhanced their growth performance. Similar results were observed in the study by Lei et al. [30], in which the addition of 0.5% *Bacillus subtilis* increased the WGR and SGR of juvenile Salmo trutta fario. Furthermore, Zokaeifar et al. [31] indicated that adding *Bacillus subtilis* at a concentration of 1.5 × 10^10^ CFU/g (0.01%) to the diet of 3.5 g Litopenaeus vannamei shrimp produced the best growth-promoting effect; however, excessive supplementation of *Bacillus subtilis* (>0.04%) did not yield the same growth-promoting benefits. It should be noted that the optimal dosage of additives varies across different studies, which may be attributed to differences in strain origin, live bacterial count, host developmental stage, basal diet composition, and rearing conditions.

This phenomenon may be attributed to the production of extracellular digestive enzymes by *Bacillus subtilis*, including proteases, amylases, and lipases that these enzymes facilitate the degradation of dietary nutrients, thereby enhancing their availability and digestibility [32]. Second, previous studies have suggested that probiotic-induced alterations in gut microbiota may influence SCFA production and host nutrient metabolism [33], such as acetic acid, propionic acid, and butyric acid promotes epithelial cell proliferation and serves as an additional energy source, helping to maintain intestinal health [34]. The enhanced growth performance observed in the present study may be associated with improved nutrient digestion and intestinal absorptive capacity induced by probiotic supplementation.

### 4.2. Effects of Bacillus subtilis on the Intestinal Digestive Enzyme Activities

The results showed that after adding *Bacillus subtilis* to the feed, the intestinal α-amylase, lipase, and trypsin activities of juvenile GIFT exhibited a trend of initially increasing and then decreasing. In the group G3 (1 × 10^9^ CFU/kg), α-amylase and lipase activities reached their highest levels (*p* < 0.05). This indicates that supplementing the diet with an appropriate amount of *Bacillus subtilis* can enhance the activity of intestinal digestive enzymes in juvenile GIFT.

Similar findings have been observed in red claw crayfish (*Cherax quadricarinatus*), as the intestinal α-amylase, lipase, and trypsin activities of red claw crayfish exhibited a trend of initially increasing and then decreasing. In the treatment group at 1.5 × 10^6^ CFU/g, α-amylase and lipase activities reached their highest levels (*p* < 0.05) [35]. Wu et al. found that dietary supplementation with *Bacillus subtilis* Ch9 significantly increased the activity of proteases, amylases, and lipases in the intestines of grass carp [36]. According to Adorian et al., the growth performance and digestive enzyme activities of Asian sea bass (*Lates calcarifer*) were significantly improved by dietary supplementation with *Bacillus subtilis* at 1 × 10^6^ CFU/g [37].

The possible reasons could be that *Bacillus bacteria* enhance intestinal enzyme activity by promoting the proliferation of beneficial microorganisms, thereby aiding in the breakdown and absorption of nutrients [38]. Wang et al. indicate that dietary supplementation with *Bacillus subtilis* of juvenile sea cucumbers (*Apostichopus japonicus*) increased the relative abundance of lactic acid bacteria in intestine [39]. Second, changes in pH can affect the activity of digestive enzymes, thereby creating an environment that is more or less conducive to their function [40]. *Bacillus subtilis* competes with gut microbiota for binding sites and receptors on the intestinal wall, and with pathogenic bacteria for attachment sites; it regulates intestinal pH to promote the stability of the microbial community, thereby enhancing digestive enzyme activity [41]. In addition, the trend of intestinal digestive enzyme activity may be related to the disruption of the gut microbiota caused by an excessive dose of *Bacillus subtilis* entering the intestine [7].

### 4.3. Effects of Bacillus subtilis on the Relative Expression Levels of Antioxidant Genes

In this study, dietary *Bacillus subtilis* supplementation significantly increased the relative expression levels of *nrf2*, *sod*, *gst*, *gsh-px* and *cat* in the livers and intestines of juvenile GIFT (*p* < 0.05), with the group G3 (1 × 10^9^ CFU/kg) showing the best performance. These results suggest that *Bacillus subtilis* supplementation influence the transcriptional regulation of antioxidant-related genes of juvenile GIFT.

The findings are consistent with previous studies. Wang et al. demonstrated that supplementing the diet with *Bacillus subtilis* upregulates the transcription of genes associated with the Nrf2-Keap1 signaling pathway in largemouth bass (*Micropterus salmoides*) that significantly increasing the expression of genes *nrf2*, *sod*, *gst* and *cat* [42]. Wang et al. indicate that addition of 1.0% of the solid-state fermentation product of *Bacillus subtilis* HGcc-1 significantly upregulated the expression of the antioxidant genes *nrf2*, *ho-1*, *cat*, *sod*, and *gst* in zebrafish (*Danio rerio*) [43]. Tang et al. demonstrated that *Bacillus subtilis* enhanced the antioxidant capacity of grass carp, manifested by the upregulation of the antioxidant enzyme-related genes *sod*, *cat*, and *gpx* [44].

This phenomenon may be due to the fact that short-chain fatty acids secreted by *Bacillus subtilis* activate G protein-coupled receptors on intestinal epithelial cells, thereby inducing a conformational change in Keap1 and reducing its ability to ubiquitinate and degrade Nrf2 [45]. Second, *Bacillus subtilis* colonizes the gut of juvenile fish, which inhibits the proliferation of harmful bacteria by secreting antimicrobial substances such as bioactive compounds and antimicrobial peptides. Furthermore, the growth and colonization of beneficial bacteria, such as lactic acid bacteria, may be enhanced, leading to a more favorable gut microbial ecosystem and a subsequent improvement in the immune status of juvenile fish. The dietary *Bacillus subtilis* supplementation in Chinese perch (*Siniperca chuatsi*), the abundance of harmful bacteria such as Enterobacteriaceae, Actinobacteria, thermophilic bacteria, and spirochetes decreased in intestines [23]. Furthermore, the liver plays a central role in maintaining redox homeostasis and regulating antioxidant responses [46]. Dietary *Bacillus subtilis* supplementation resulted in increased hepatic nrf2 expression, suggesting that the transcriptional regulation of antioxidant-related genes may have been influenced by the dietary treatment.

### 4.4. Effects of Bacillus subtilis on the Relative Expression Levels of Inflammatory Genes

The results showed that dietary *Bacillus subtilis* supplementation significantly downregulated the relative expression levels of *tnf-α*, *il-iβ*, *il-8* and significantly upregulated the relative expression levels of the *il-10* and *tgf-β1* genes (*p* < 0.05), with the group G3 (1 × 10^9^ CFU/kg) showing the best performance. It suggest that *Bacillus subtilis* may regulate immune-related gene expression in juvenile GIFT.

Tang et al. demonstrated that the *Bacillus subtilis* improved the immune response in grass carp, as evidenced by the upregulation of the anti-inflammatory cytokine *il-10* and downregulation of the pro-inflammatory cytokines *tnf-α*, *il-1β*, and *il-8* [44]. Hou et al. indicate that the *Bacillus subtilis* upregulated the expression of the *il-10* and *tgf-β1* genes in the intestines of darkbarbel catfish (*Pelteobagrus fulvidraco*) and downregulated the expression of the *tnf-α* and *il-1β* genes [47]. Unlike this experiment, He et al. demonstrated that the *Bacillus subtilis* C-3102 increased the expression of the *tnf-α* and *il-1β* genes in the intestine [48]. This may be related to differences in strains and farmed species.

The improvement in the immune function of juvenile fish may be attributable to *Bacillus subtilis* stimulating both the host’s nonspecific and specific immune responses. Studies have shown that *Bacillus subtilis* can influence in the transcription of multiple genes involved in innate and adaptive immune responses, thereby enhancing the host’s immunity [49]. Second, *Bacillus subtilis* secretes subtilisin, which is a key factor in inducing the production of IL-10 [50]. In addition, short-chain fatty acids secreted by *Bacillus subtilis* can modulate downstream signaling pathways via G protein-coupled receptors (GPCRs), thereby directly regulating the immune response of intestinal epithelial cells (IECs) [51]. Butyric acid has been shown to improve gut immune function by modulating the interactions between the host and microorganisms [52]. It should be noted that only gene transcription levels were measured in the present study.

### 4.5. Effects of Bacillus subtilis on the Gut Microbiome

Dietary *Bacillus subtilis* supplementation was associated with changes in the intestinal microbial composition of juvenile GIFT. Compared with the control group (G0), the relative abundance of Proteobacteria tended to decrease, whereas the abundances of Firmicutes, Bacteroidota, and Fusobacteriota increased in several supplemented groups. In particular, the abundance of Firmicutes increased from 1.07% in G0 to 22.91% in G3 (1 × 10^9^ CFU/kg), while Bacteroidota increased from 3.02% to 22.52%. Previous studies have reported associations between Firmicutes and Bacteroidota and host nutrient utilization, immune regulation, and intestinal homeostasis [53]. Similarly, Tachibana et al. reported that dietary supplementation with *Bacillus subtilis* and *Bacillus licheniformis* increased the relative abundance of Firmicutes in Nile tilapia [54]. Proteobacteria is commonly one of the dominant bacterial phyla in the intestinal microbiota of fish [55]. In the present study, the ratio of (Firmicutes + Bacteroidota + Fusobacteriota)/Proteobacteria increased following *Bacillus subtilis* supplementation. Although this ratio has been proposed as a microbial community indicator in some studies [56], its biological significance should be interpreted cautiously because it does not directly measure host health status. The observed shifts in microbial composition may be associated with the improved growth performance observed in the supplemented groups.

Alpha-diversity analysis showed a tendency toward higher microbial richness in the *Bacillus subtilis*-supplemented groups, particularly in G3 and G5, although no statistically significant differences were detected among treatments. Similarly, PCoA revealed partial clustering of samples according to dietary treatments. However, the PERMANOVA analyses indicated that the overall beta diversity differences among groups were not statistically significant. Therefore, the present results suggest that *Bacillus subtilis* supplementation may influence specific bacterial taxa without causing a significant restructuring of the overall intestinal microbial community. The G3 group exhibited the highest number of unique OTUs and relatively high diversity indices, suggesting that moderate *Bacillus subtilis* supplementation may support a more diverse microbial community. Similar dose-dependent effects of *Bacillus* supplementation on intestinal microbiota have been reported in Anguilla japonica and Micropterus salmoides [45,57]. Excessive probiotic supplementation has been suggested to alter microbial interactions and potentially affect community stability [30,58].

PICRUSt2-based functional prediction suggested differences in the predicted functional potential of the intestinal microbiota among treatments. At KEGG Level 2, pathways related to amino acid metabolism, carbohydrate metabolism, energy metabolism, lipid metabolism, and cofactor and vitamin metabolism showed variations in predicted relative abundance among groups. Lipid and amino acid metabolic pathways can effectively enhance the digestive and absorptive functions of animals, thereby improving their growth performance [59]. Chen et al. studies on the *Cherax quadricarinatus* demonstrated that supplementation with *Bacillus subtilis* upregulated pathways related to amino acid and energy metabolism in the red-clawed crayfish, and increased the activity of α-amylase and trypsin [35]. At KEGG Level 3, pathways associated with biotin metabolism, folate biosynthesis, pantothenate and CoA biosynthesis, and other cofactor- and vitamin-related functions exhibited higher predicted relative abundances in G3 and G4. In addition, pathways associated with energy metabolism showed higher predicted relative abundance in G3, whereas bacterial chemotaxis displayed a lower predicted relative abundance in G5. Folate and thiamine are micronutrients essential for fish growth, energy metabolism and immune cell function [55]. It is suggested that an appropriate amount of *Bacillus subtilis* may have improved the vitamin metabolism capacity of juvenile GIFT.

## 5. Conclusions

In conclusion, under the experimental conditions of the present study, dietary *Bacillus subtilis* supplementation influenced growth performance, feed utilization, digestive enzyme activity, the transcription of antioxidant and immune genes, and the intestinal microbial community of juvenile GIFT (*Oreochromis niloticus*). Among the tested supplementation levels, the 1 × 10^9^ CFU/kg treatment generally exhibited favorable responses across multiple indicators. Collectively, these results suggest that dietary *Bacillus subtilis* may contribute to physiological regulation and modulation of the intestinal microbial ecosystem in juvenile GIFT under normal culture conditions. Furthermore, only transcriptional responses were evaluated, whereas antioxidant enzyme activities, protein expression, and disease resistance were not directly assessed. Because a dose–response optimization analysis was not performed, the biological optimum supplementation level remains to be further validated. Moreover, PICRUSt2 results represent predicted functional potential rather than directly measured metabolic activity. The present study was conducted under controlled experimental conditions. Further investigations under commercial farming environments and pathogen challenge models are required to validate the long-term efficacy of dietary *Bacillus subtilis* supplementation.

## Figures and Tables

**Table 1:** Composition of the experimental diets for juvenile GIFT (g/kg of dried feed).

Ingredients		*Bacillus subtilis* Levels (CFU/kg)				
	G0	G1	G2	G3	G4	G5
*Bacillus subtilis*	0	0.93 ± 0.12 × 10^7^	0.98 ± 0.21 × 10^8^	1.1 ± 0.17 × 10^9^	1.2 ± 0.27 × 10^10^	1.13 ± 0.18 × 10^11^
Soybean oil	30.00	30.00	30.00	30.00	30.00	30.00
Fish oil	10.00	10.00	10.00	10.00	10.00	10.00
Fish meal	80.00	80.00	80.00	80.00	80.00	80.00
Rapeseed meal	220.00	220.00	220.00	220.00	220.00	220.00
Soybean meal	330.00	330.00	330.00	330.00	330.00	330.00
Dextrin	240.00	240.00	240.00	240.00	240.00	240.00
Gelatin	50.00	50.00	50.00	50.00	50.00	50.00
Vitamin mixture ^1^	10.00	10.00	10.00	10.00	10.00	10.00
Mineral mixture ^2^	10.00	10.00	10.00	10.00	10.00	10.00
Choline chloride	5.00	5.00	5.00	5.00	5.00	5.00
Sodium chloride	5.00	5.00	5.00	5.00	5.00	5.00
Adhesive	5.00	5.00	5.00	5.00	5.00	5.00
Attractant	0.1	0.1	0.1	0.1	0.1	0.1
Preservative	1.0	1.0	1.0	1.0	1.0	1.0
Antioxidants	0.2	0.2	0.2	0.2	0.2	0.2
Crude protein	34.00	34.00	34.00	34.00	34.00	34.00
Crude fat	7.35	7.35	7.35	7.35	7.35	7.35
Ash	2.94	2.93	2.95	2.96	2.94	2.96
Moisture	9.36	9.36	9.36	9.36	9.36	9.36
Gross energy (Mcal/kg)	3.76	3.76	3.76	3.76	3.76	3.76

^1^ Vitamin mixture (IU or mg/kg of dried feed): vitamin A 2500 IU; vitamin D_3_ 1200 IU; vitamin K_3_ 60 IU; folic acid 5 mg; vitamin B_1_ 10 mg; vitamin B_2_ 10 mg; vitamin B_6_ 20 mg; vitamin B_12_ 0.15 mg; niacin 40 mg; calcium pantothenate 20 mg; inositol 150 mg; biotin 0.2 mg; vitamin C 150 mg; vitamin E 60 mg. ^2^ Mineral mixture (mg/kg of dried feed): iron 15 mg; zinc 20 mg; manganese 2 mg; copper 1 mg; iodine 0.2 mg; selenium 0.05 mg; cobalt 0.25 mg; magnesium 0.06 mg; potassium 40 mg.

**Table 2:** Primer sequences for RT-PCR of genes in the juvenile GIFT.

Gene	Primer Sequence (5′→3′)	Amplicon Size (bp)	Tm (°C)	Gene Bank
*β-actin* ^1^	F: AAGGACCTGTACGCCAACAC	196	60	KJ126772.1
	R: ACATCTGCTGGAAGGTGGAC			
*sod* ^2^	F: GTCTGCTGTTACGGTGGCTGTAC	82	60	XM_003449940.5
	R: ATCAATGCGAAGTCTTCCACTGTCC			
*cat* ^3^	F: GCCCAGCTCTTCATCCAGAAACG	107	60	XM_003447521.5
	R: TTGGCCTCCGCATTGTACTTCTTG			
*gpx* ^4^	F: AAAATGTGGCGTCTCTCTGAGGAAC	85	60	NM_001279711.1
	R: AGACCTTCGGCGGAGTAGCG			
*gst* ^5^	F: CACCCCAGATCCCAAACCCAAAC	140	60	XM_025897213.1
	R: CAACAAGCAGCACATCAGCAAGG			
*nrf2* ^6^	F: ACTCGGACATGGAGGAGATGG	131	60	XM_003447296.5
	R: TGGTTGCTTAGATGGTGTGGATAC			
*tnf-α* ^7^	F: AAGCCTCACAATTCTCAGCCACAG	114	60	NM_001279533.1
	R: AAGCTATCCTGAATGGTGTCTTGGC			
*tgf-β1* ^8^	F: TGCCTCCTCTCCACTGAGTGATTC	80	60	NM_001311325.1
	R: CTCCTCCGACTTCCCTTTCAATGC			
*il-1β* ^9^	F: AAAATGTCGCGGTACATCCC	131	60	XM_019365844.2
	R: TGCTCCAGAAACAGCACAAC			
*il-6* ^10^	F: CCCCGCCTCTATCCCGAAGC	91	60	XM_019350387.2
	R: ACCGAGTAGATGAGCAGACCCTTG			
*il-8* ^11^	F:ACCGGGAGCTGAAAGAAAGACAATC	88	60	NM_001279704.1
	R:TCCGAAAGTAGATGAAGCCTGAAGC			
*il-10* ^12^	F: AGCAGCAGGAGCATCAGCATTTC	110	60	XM_013269189.3
	R: ACGACAAGGAGGACGGTCTG			

Note: F: forward primer. R: reverse primer. ^1^ *β-actin*: non-regulated reference gene. ^2^ *sod*: superoxide dismutase. ^3^ *cat*: catalase. ^4^ *gpx*: glutathione peroxidase. ^5^ *gst*: glutathione S-transferases. ^6^ *nrf2*: erythroid-derived 2. ^7^ *tnf-α*: tumor mor necrosis factor α. ^8^ *tgf-β1* transforming growth factor-β. ^9^ *il-1β*: interleukin-1β. ^10^ *il-6*: interleukin-6. ^11^ *il-8*: interleukin-8. ^12^ *il-10*: interleukin-10.

**Table 3:** The effects of *Bacillus subtilis* on growth performance of tilapia GIFT juvenile.

Index	Treatments ^1^						*p*-Value
	G0	G1	G2	G3	G4	G5	
Final weight (g)	89.17 ± 1.72 ^c^	99.73 ± 1.87 ^b^	102.71 ± 6.11 ^b^	115.01 ± 3.24 ^a^	107.27 ± 4.62 ^b^	105.43 ± 6.01 ^b^	0.000
WGR ^2^ (%)	451.45 ± 10.65 ^c^	516.74 ± 11.55 ^b^	535.19 ± 37.80 ^b^	611.28 ± 20.06 ^a^	563.37 ± 28.56 ^b^	552.01 ± 37.20 ^b^	0.000
FCR ^3^	1.92 ± 0.05 ^a^	1.68 ± 0.04 ^b^	1.63 ± 0.12 ^b^	1.42 ± 0.05 ^c^	1.54 ± 0.08 ^bc^	1.57 ± 0.11 ^b^	0.000
SGR ^4^ (%/day)	2.85 ± 0.04 ^c^	3.03 ± 0.03 ^b^	3.08 ± 0.10 ^b^	3.27 ± 0.05 ^a^	3.15 ± 0.08 ^ab^	3.12 ± 0.09 ^b^	0.000
DGI ^5^ (%/day)	3.23 ± 0.05 ^c^	3.51 ± 0.05 ^b^	3.59 ± 0.16 ^b^	3.89 ± 0.08 ^a^	3.70 ± 0.11 ^ab^	3.66 ± 0.15 ^b^	0.000
SR ^6^ (%)	93.33 ± 1.67 ^b^	96.67 ± 1.67 ^ab^	96.67 ± 1.67 ^ab^	100 ± 0.00 ^a^	100 ± 0.00 ^a^	98.33 ± 1.67 ^a^	0.017

Note: All the data are presented as mean ± SD (*n* = 3). According to the LSD test, the mean values marked with different superscript letters in the same row are significantly different from each other (*p* < 0.05). ^1^ Treatments: a basal diet without any supplementation (G0) and a basal diet supplemented with 1 × 10^7^ (G1), 1 × 10^8^ (G2), 1 × 10^9^ (G3), 1 × 10^10^ (G4), 1 × 10^11^ (G5) CFU/kg *Bacillus subtilis* of the diet. ^2^ WGR: weight gain rate. ^3^ FCR: feed conversion rate. ^4^ SGR: specific growth rate. ^5^ DGI: daily growth index. ^6^ SR: survival rate.

**Table 4:** The effects of *Bacillus subtilis* on digestive enzymes in the intestines of tilapia GIFT juvenile.

Index	Treatments ^1^					
	G0	G1	G2	G3	G4	G5
Trypsin (U/mgprot)	2356.23 ± 272.76 ^ab^	2459.20 ± 248.97 ^ab^	2345.71 ± 119.27 ^ab^	2634.82 ± 89.70 ^a^	2345.95 ± 84.99 ^ab^	2205.70 ± 75.65 ^b^
α-amylase (U/mgprot)	478.54 ± 37.94 ^b^	552.47 ± 28.38 ^b^	556.84 ± 64.95 ^b^	662.35 ± 50.00 ^a^	525.15 ± 24.72 ^b^	513.55 ± 65.65 ^b^
Lipase (U/gprot)	241.82 ± 29.50 ^d^	265.66 ± 15.53 ^d^	390.96 ± 19.35 ^ab^	417.22 ± 16.86 ^a^	364.88 ± 15.18 ^b^	325.06 ± 14.73 ^c^

Note: All the data are presented as mean ± SD (*n* = 3). According to the LSD test, the mean values marked with different superscript letters in the same row are significantly different from each other (*p* < 0.05). ^1^ Treatments: A basal diet without any supplementation (G0) and a basal diet supplemented with 1 × 10^7^ (G1), 1 × 10^8^ (G2), 1 × 10^9^ (G3), 1 × 10^10^ (G4), 1 × 10^11^ (G5) CFU/kg *Bacillus subtilis* of the diet.

## Data Availability

The data that support the findings of this study are available from the corresponding authors upon reasonable request.

## Supplementary Materials

The following supporting information can be downloaded at: https://www.mdpi.com/article/10.3390/ani16131942/s1, Figure S1. Distances from individual samples to their respective group centroids based on Bray-Curtis dissimilarity. Each point represents one biological replicate (*n* = 3 per group). Boxes indicate the median and interquartile range. PERMDISP analysis revealed no significant differences in multivariate dispersion among treatment groups (F = 1.1181, P = 0.387, 999 permutations).
