# Supplementary material for: Dietary Bacillus subtilis Improves Growth Performance, Digestive Enzyme Activity, Antioxidant and Inflammatory Responses, and Gut Microbiota in Juvenile GIFT (Oreochromis niloticus)
Source: Animals (Basel). 2026 Jun 23;16(13):1942. doi: 10.3390/ani16131942 (PMC13360612; doi:10.3390/ani16131942)
Supplement: Supplementary file 1 [file animals-16-01942-s001.zip › code.pdf]

Beta-diversity and PERMDISP analysis based on G_OTU.xlsx. (R version 4.6.0; R(vegan) version 2.7-5)

```
library(readxl)
library(vegan)
library(ggplot2)

set.seed(123)
```

1. Read OTU table

```
otu <- read_excel("G_OTU.xlsx")
```

2. Extract only sample tag-count columns

```
tag_cols <- grep("^G[0-5]-[1-3]_tags$", colnames(otu), value = TRUE)

otu_table <- as.data.frame(otu[, c("OTU", tag_cols)])
```

3. Set OTU IDs as row names

```
rownames(otu_table) <- otu_table$OTU
otu_table$OTU <- NULL
```

4. Convert all abundance columns to numeric

```
otu_table[] <- lapply(otu_table, as.numeric)
```

5. Transpose table: samples as rows, OTUs as columns

```
otu_t <- t(otu_table)
```

6. Define sample names and groups automatically

```
sample_names <- rownames(otu_t)

group <- factor(
  sub("-[1-3]_tags$", "", sample_names),
  levels = c("G0", "G1", "G2", "G3", "G4", "G5")
)

metadata <- data.frame(
  Sample = sample_names,
  Group = group
)

print(metadata)
```

7. Calculate Bray-Curtis distance

```
bc.dist <- vegdist(otu_t, method = "bray")
```

8. PERMANOVA

```
permanova_result <- adonis2(
  bc.dist ~ group,
  permutations = 999
)

print(permanova_result)
```

9. ANOSIM

```
anosim_result <- anosim(
  bc.dist,
  group,
  permutations = 999
)

print(anosim_result)
```

10. PERMDISP

```
disp <- betadisper(
  bc.dist,
  group
)

permdisp_result <- permutest(
  disp,
  permutations = 999
)

print(permdisp_result)
```

11. Extract distance-to-centroid values

```
dist_df <- data.frame(
  Sample = sample_names,
  Group = group,
  Distance = disp$distances
)

print(dist_df)
```

12. Plot distance-to-centroid boxplot

```
p <- ggplot(dist_df, aes(x = Group, y = Distance, fill = Group)) +
  geom_boxplot(width = 0.6, outlier.shape = NA, alpha = 0.7) +
  geom_jitter(width = 0.1, size = 3) +
```

```
  theme_classic(base_size = 16) +
  labs(
    x = "Treatment group",
    y = "Distance to group centroid"
  ) +
  theme(
    legend.position = "none"
  )

print(p)
```

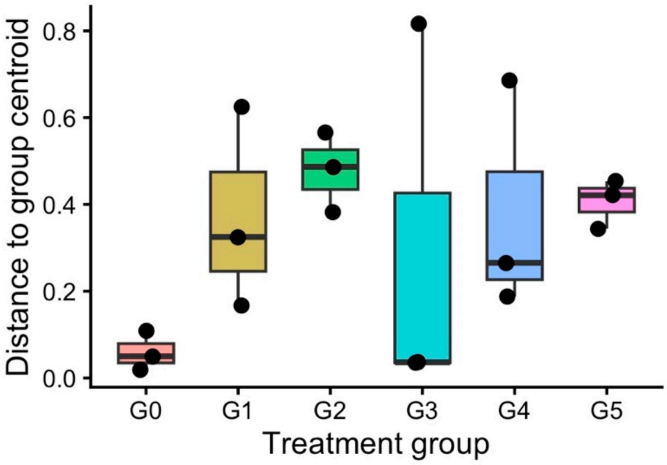

Figure S1. Distances from individual samples to their respective group centroids based on Bray-Curtis dissimilarity. Each point represents one biological replicate (n = 3 per group). Boxes indicate the median and interquartile range. PERMDISP analysis revealed no significant differences in multivariate dispersion among treatment groups (F = 1.1181, P = 0.387, 999 permutations).
